# Anatomical Study of the Variants of the Extrapelvic Part of the Pudendal Nerve

**DOI:** 10.7759/cureus.28281

**Published:** 2022-08-22

**Authors:** Rajiv Ranjan, Camellia Chanda, Rajesh Kushwaha, Alka Rashmi Nag

**Affiliations:** 1 Anatomy, Rajendra Institute of Medical Sciences, Ranchi, IND; 2 Anatomy, Shaheed Nirmal Mahto Medical College, Dhanbad, IND

**Keywords:** sciatic nerve, sacrospinous ligament, pudendal nerve, nerve entrapment, anomalous communication, anatomical variation

## Abstract

Background

A comprehensive understanding of the anatomy of the extra pelvic course of the pudendal nerve and its variations is crucial when undertaking perineal and perirectal procedures to safeguard the integrity of the extrapelvic segment of the pudendal nerve and its branches. So we aimed to identify the changes in the pudendal nerve's extrapelvic branching pattern before it enters the pudendal canal and its relationships and connections.

Materials and Methods

A cross-sectional descriptive study was carried out on 26 formalin embalmed adult human cadavers between 20 to 65 years (16 male and 10 female) of north Indian origin. Anatomical course, variations, and connections of the pudendal nerve before entering the pudendal canal were noted.

Results

The extrapelvic course of the pudendal nerve was examined in 52 hemipelves (26 cadavers) after meticulous dissection. Single pudendal nerve trunk (type I) was identified in 51.9% of hemipelves. Two trunked pudendal nerve with inferior gluteal nerve piercing the sacrospinous ligament (type IIa) was observed in 13.5% of hemipelves. 23.1% of hemipelves exhibited two trunked pudendal nerves with inferior gluteal nerve not piercing the sacrospinous ligament(type IIb). Three trunked pudendal nerve (type III) was observed in 11.5% of hemipelves. In 14/52 hemipelves (26.9%), communication with the sciatic nerve was noted, whereas, in 38/52 hemipelves (73.1%), no communication with the sciatic nerve was present.

Conclusion

The extrapelvic course of the pudendal nerve may present with an earlier subdivision or even an aberrant connection with the sciatic nerve. These anatomical variations of the extra pelvic course of the pudendal nerve, its variations, and connections are essential for all surgeons and anesthetists operating in the perineal and perirectal region to avoid unwanted complications.

## Introduction

The pudendal nerve develops right above the superior border of the sacrotuberous ligament and the upper fibers of the ischiococcygeus. It is derived from the ventral divisions of the second, third, and fourth sacral ventral rami. It leaves the pelvis through the greater sciatic foramen to descend posterior to the sacrospinous ligament. It accompanies the internal pudendal artery through the lesser sciatic foramen into the pudendal canal on the lateral wall of the ischioanal fossa. It passes anterior to the lateral third of the sacrotuberous ligament and medial or posterior to the ischial spine before entering the perineum through the lesser sciatic foramen via the pudendal canal, also known as Alcock's canal after the prominent Irish anatomist Benjamin Alcock [1]. The pudendal nerve gives rise to three main branches: the inferior rectal, perineal, and dorsal nerve of the clitoris or the penis. The pudendal nerve supplies motor and sensory innervation to the perineum [2].

Sound knowledge of the anatomical variations of the pudendal nerve and its branches is essential for all surgeons performing perineal surgeries such as sacrospinous colpopexy, better known as Richter's procedure [3]. The procedure treats vaginal and uterine prolapse and the tension-free vaginal tape to treat female stress urinary incontinence [4,5]. A good understanding of the course, branching pattern, and variations of the pudendal nerve are helpful in perineal/perirectal operative procedures such as external urethral sphincter repair or drainage of complex perirectal abscesses [6].

In the gluteal region, the pudendal nerve lies posterior to the sacrospinous ligament and anterior to the sacrotuberous ligament. The relationship of the pudendal nerve to the sacrospinous ligament has significant clinical ramifications. However, there is a lack of literature examining the variations in pudendal nerve anatomy in the gluteal region; as the pudendal nerve passes around the ischial spine and crosses behind the sacrospinous ligament, it is of significance during several gynecological procedures. For example, inaccurate placement of sacrospinous sutures during a sacrospinous fixation for vaginal prolapse, either through the entire thickness of the ligament or too far laterally, might cause pudendal nerve entrapment [3]. Therefore, to protect the integrity of the pudendal nerve and its branches, a clear understanding of the anatomy of the pudendal nerve and its variations is essential while performing perineal or perirectal procedures. Henceforth, the present study was conducted with the Primary objective of ascertaining the incidence of the early divisions of the pudendal nerve proximal to the pudendal canal with emphasis on its clinical relevance. A secondary objective was identifying variations in its extrapelvic course, particularly aberrant connections bearing embryological significance and clinical applicability.

## Materials and methods

The study was conducted in the Department of Anatomy, Rajendra Institute of Medical Sciences (RIMS), Ranchi, Jharkhand, India, after the Institutional Ethics Committee (IEC) of RIMS, Ranchi having approval number 242 Dated 03/06/2021. A cross-sectional descriptive study was carried out over 24 months on 26 (16 male and 10 female) formalin embalmed adult human cadavers of north Indian ethnicity aged between 20 to 65 years. The cadavers with any apparent perineal/perianal anomaly were excluded from this study. The pudendal nerve was exposed through a posterior dissection approach with the cadaver in a prone position. The skin between the anterior iliac spine, the greater trochanter, the ischial tuberosity, and the posterior superior iliac spine was removed. The gluteus maximus was exposed, sectioned longitudinally near its origin, and was reflect­ed laterally to its insertion [7]. The gluteus medius and minimus, piriformis, two Gemelli, tendon of obturator internus, and quadratus femoris were identified under its cover. The sciatic, superior, and in­ferior gluteal nerves and vessels were cleaned and noted. Eventually, the attachments of the sacrotuberous ligament were dissected and detached from their origin to trace the pudendal nerve, internal pudendal vessels, and the nerve to the obturator internus. Its relation with the sciatic nerve and the sacrotuberous ligament was observed and noted. The sacral spine and the sacrospinous ligament were dissected and noted.

The presence or absence of early subdivision of the pudendal nerve was noted. Also noted is whether such variations are unilateral or bilateral. This study classified the variation in the trunk of the extra pelvic part of the pudendal nerve into four types. This was based on the classification proposed by Mahakkanukrauh et al. (2005) with obvious modifications [8]. Type I includes Single trunked pudendal nerve (Normal variant, passing posterior to the sacrospinous ligament without any division/communication to the sciatic nerve) [1]. Type IIa includes double trunked (bifurcated) pudendal nerve with one trunk as inferior rectal nerve piercing/passing through the sacrospinous ligament or fixed to the sacral spine by ligamentous strands. Type IIb includes double trunked (bifurcated) pudendal nerve with one inferior rectal nerve not piercing/passing through the sacrospinous ligament. Type III includes the triple trunked (trifurcated) pudendal nerve. Moreover, anomalous communication between the pudendal nerve and the sciatic nerve in the variants mentioned above was also noted, if present.

The variations of the pudendal nerve; in its trunks, connections, and branching patterns were photographed by the camera of the Motorola one fusion plus, a 64MP Samsung ISOCELL Plus GW1 1/1.72" sensor with 0.8µm pixels and f/1.8 lens, under a light source [9].

Descriptive data were analyzed and interpreted using Jamovi software for Windows, Version 2.2.5.0 [10]. A chi-square test of independence was conducted between gender, side of the limb, and sciatic nerve communication status. Fisher's Exact Test was used if 50% of cells had an expected count of less than 5. When the P value was found to be more than 0.05, it was not considered statistically significant. It was considered statistically significant if the P value was less than 0.05.

## Results

**Figure 1:**
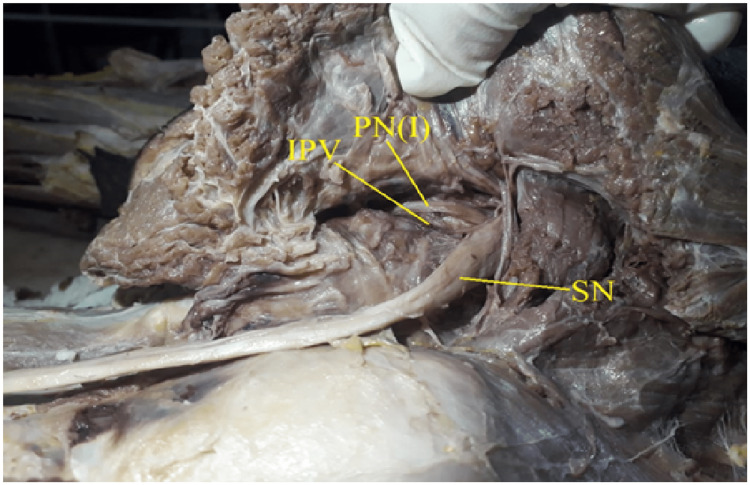
Showing type I pattern of Pudendal nerve: single trunk, with no sciatic communication. PN(I): Pudendal nerve type I, IPV: Internal Pudendal Vessels, SN: Sciatic Nerve

**Figure 2:**
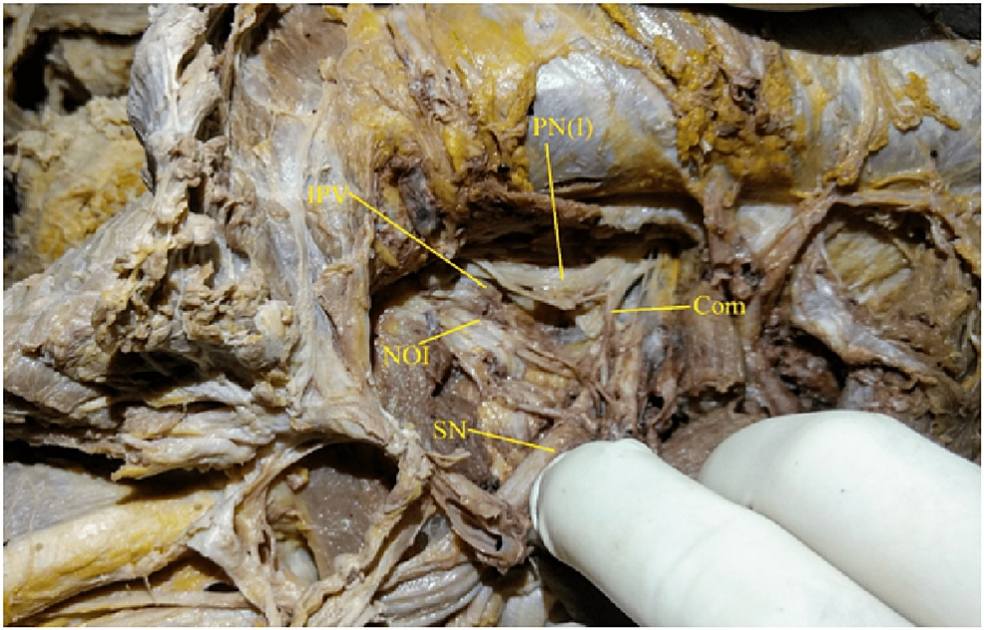
Showing type I pattern of Pudendal nerve: single trunk, with sciatic communication. PN(I): Pudendal nerve, IPV: Internal Pudendal Vessels, NOI: Nerve to Obturator Internet, SN: Sciatic Nerve, Com: Sciatic communicating branch

The present study investigated the pudendal nerve trunk related to the sacrospinous ligament in 26 cadavers (16 males and 10 females), or 26 right and left hemipelves. In the present study, 27 out of 52 hemipelves (51.9%) were identified as type I having a single pudendal nerve trunk (Fig 1, 2).

**Figure 3:**
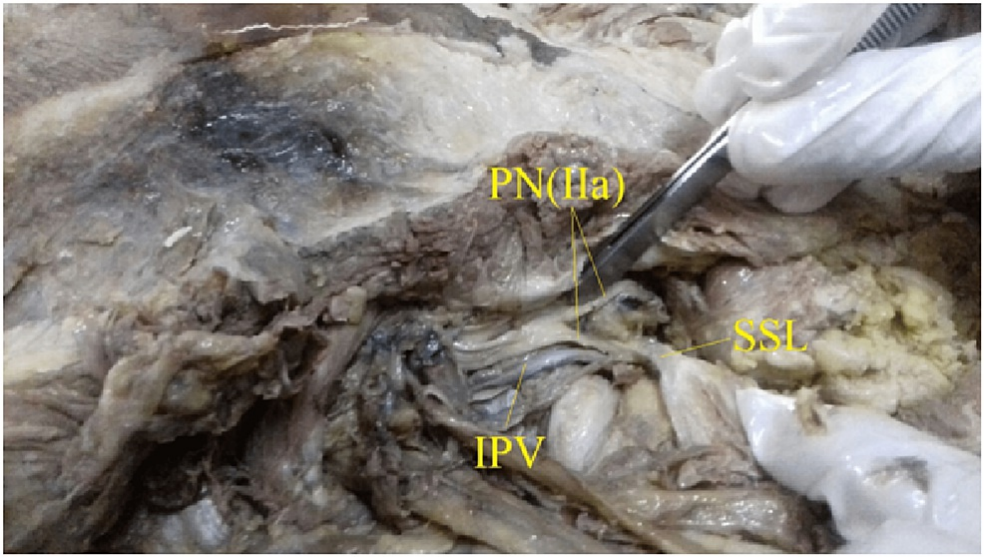
Showing type IIa, double trunked with inferior rectal nerve piercing the sacrospinous ligament. PN(IIa): Bifurcated Pudendal nerve with inferior rectal nerve piercing the sacrospinous ligament. IPV: Internal Pudendal Vessels SSL: SSL: Sacrospinous ligament

Type IIa (Fig 3), Exhibits double trunks of the pudendal nerve; one trunk was the inferior rectal nerve and was found piercing/passing through the sacrospinous ligament. This branching pattern was observed in 7 out of 52 hemipelves (13.5%).

**Figure 4:**
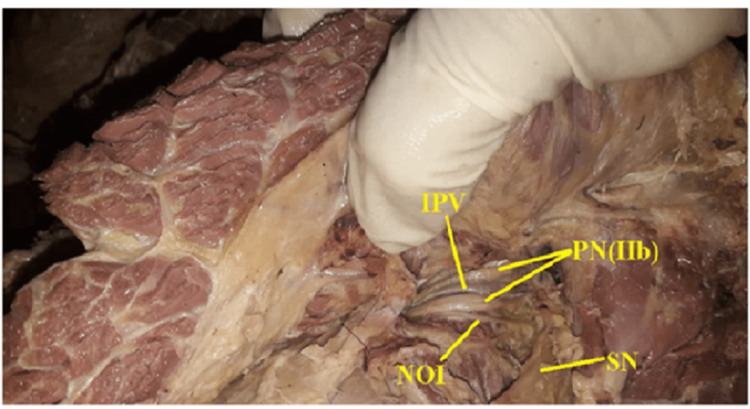
Showing type II b, double trunked pudendal nerve with inferior rectal nerve not piercing the sacrospinous ligament, no communication with Sciatic nerve. PN(IIb): Bifurcated Pudendal nerve, IPV: Internal Pudendal Vessels, NOI: Nerve to Obturator Internus, SN: Sciatic Nerve

**Figure 5:**
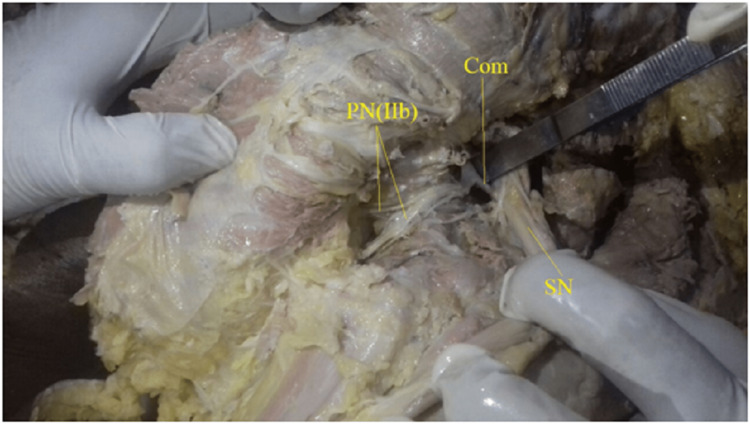
Showing type II b, two trunked pudendal nerves with inferior rectal nerve not piercing the sacrospinous ligament, communication with Sciatic nerve present. PN(IIb): Bifurcated Pudendal nerve, SN: Sciatic Nerve, Com: Sciatic communicating branch

Twelve out of 52 hemipelves (23.1%) were type IIb (Fig 4, 5), where bifurcated trunks of the pudendal nerve were observed, but the inferior rectal nerve did not pass through the sacrospinous ligament.

**Figure 6:**
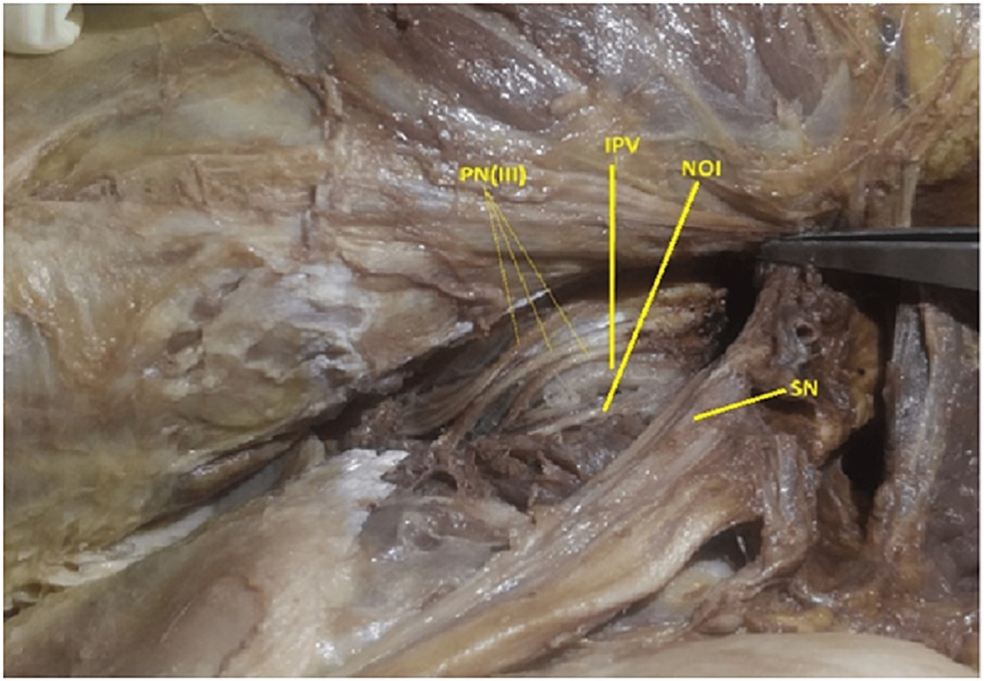
Showing type III, Triple trunked pudendal nerve, no sciatic nerve communication. PN(III): Trifurcated Pudendal nerve, IPV: Internal Pudendal Vessels, NOI: Nerve to Obturator Internet, SN: Sciatic Nerve

**Figure 7:**
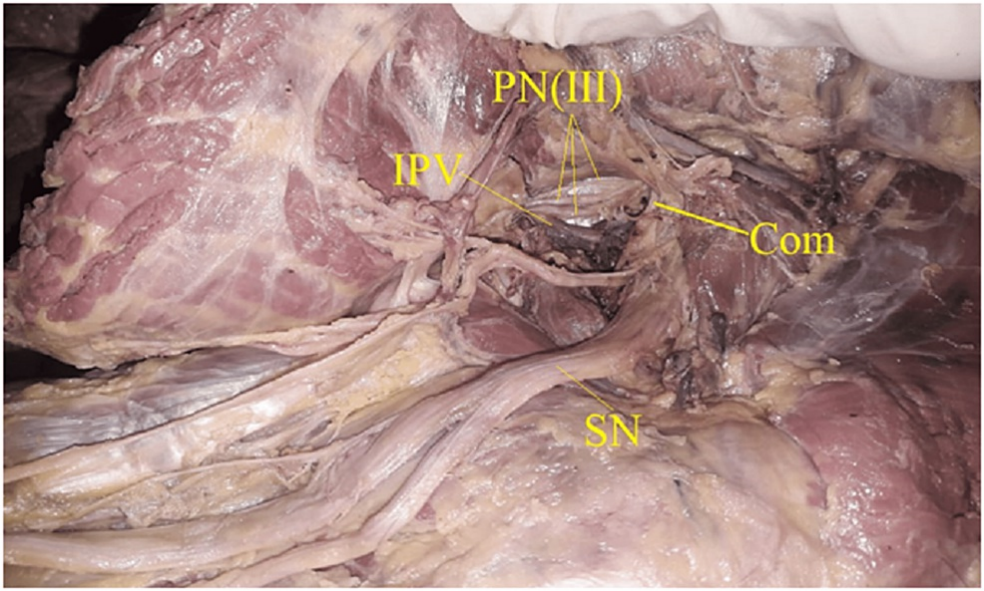
Showing type III, Triple trunked pudendal nerve, with sciatic nerve communication. PN(III): Trifurcated Pudendal nerve, IPV:Internal Pudendal Vessels, SN: Sciatic Nerve, Com: Sciatic communicating branch

Six out of 52 hemipelves (11.5%) were type III (Fig 6, 7) where trifurcated trunks of pudendal nerve were observed.

Fourteen out of 52 hemipelves (26.9%) exhibited a communicating branch connecting the pudendal nerve with the sciatic nerve, whereas in 38 out of 52 hemipelves (73.1%), no such communication was observed (Table 1).

**Table 1:** Showing the distribution of pudendal nerve variant type and presence of sciatic communication Type I: single trunked pudendal nerve (Normal variant with no subdivision), Type IIa: double trunked pudendal nerve with one trunk as an inferior rectal nerve piercing/passing through the sacrospinous ligament Type IIb: double trunked pudendal nerve with one as an inferior rectal nerve not piercing through the sacrospinous ligament Type III: triple trunked pudendal nerve

Type	Yes	No	Total
I	6(11.54%)	21(40.38%)	27(51.92%)
IIa	0(0%)	7(13.46%)	7(13.46%)
IIb	5(9.62%)	7(13.46%)	12(23.08%)
III	3(5.77%)	3(5.77%)	6(11.54%)
Total	14(26.92%)	38(73.08%)	52(100%)

A chi-square test of independence was conducted between gender, side of the limb,, and sciatic nerve communication status. All expected cell frequencies were greater than five. There was statistically no significant association between gender and sciatic nerve communication status, χ2(1) = 0.061, p = 0.805. Also, there was statistically no significant association between the laterality, i.e., left or right limb, and sciatic nerve communication status, χ2(1) = 3.52, p = 0.061 (table 2). A chi-square test of independence was conducted between the pudendal nerve variant type and sciatic nerve communication status. Fisher's Exact Test was applied as 50% of cells have an expected count of of less than 5. There was statistically no significant association between the pudendal nerve variant type and sciatic nerve communication status (two-tailed p = 0.099) (table 2).

**Table 2:** Showing the association between Gender, Laterality, Pudendal nerve variants subtypes, and Sciatic communication

Sciatic communication			P-Value
Gender	Yes	No	P-value: 0.805
Male	9(17.3%)	23(44.23%)	
Female	5(9.62%)	15(28.85%)	
Total	14(26.92%)	38(73.08%)	
Side of the limb			P-value: 0.061
Sides	Yes	No	
Right	4(7.7%)	22(42.31%)	
Left	10(19.23%)	16(30.77%)	
Total	14(26.92%)	38(73.08%)	
Pudendal nerve variant type			P-value: 0.099
Types of Variants	Yes	No	
I	6(11.54%)	21(40.38%)	
IIa	0(0%)	7(13.46%)	
IIb	5(9.62%)	7(13.36%)	
III	3(5.77%)	3(5.77%)	
Total	14(26.92%)	38(73.08%)	

A chi-square test of independence was conducted between the gender, laterality, and the pudendal nerve variant type. Fisher's Exact Test was applied as 50% or more cells have an expected count of less than 5. There was a statistically significant association between the pudendal nerve variant type and gender, Fisher's Exact Test (two-tailed p = 0.034). In contrast, there was statistically no significant association between the pudendal nerve variant type and laterality, i.e., right or left side of the limb (two-tailed p = 0.64) (table 3).

**Table 3:** Showing the association between the Gender, Laterality with Pudendal nerve variant subtype. *Statistically Significant

Type	Male	Female	Total
I	19(36.5%)	8(15.5%)	27(52%)
IIa	1(1.9%)	6(11.5%)	7(13.5%)
IIb	7(13.5%)	5(9.6%)	12(23%)
III	5(9.6%)	1(1.9%)	6(11.5%)
Total	32(61.5%)	20(38.5%)	52(100%)
Fisher’s Exact Test value:8.126 df: 3 P-value: 0.034*			
Right Limb/Left limb			
Type	Right	Left	Total
I	15(28.8%)	12(23%)	27(52%)
IIa	4(7.7%)	3(5.8%)	7(13.5%)
IIb	4(7.7%)	8(15.4%)	12(23%)
III	3(5.8%)	3(5.8%)	6(11.5%)
Total	26(50%)	26(50%)	52(100%)
Fisher’s Exact Test value:1.90 ,df: 3 , P-value: 0.64			

## Discussion

The present study depicts the variations in the trunk of the pudendal nerve into four types. A comparison was made regarding the present study's findings with the previous stud­ies reporting various patterns in divisions of the pudendal nerve trunk (Table 4).

**Table 4:** Comparison table with the previous studies

Study	Mahakkanukrauh et al. (2005)[8]	Kocabiyik et al. (2008)[11]	Pi­rro et al. (2009)[12]	Matejcik et al. (2012) [13]	Maldonado et al. (2015)[14]	Present study (2022)
Single trunk (I)	56.2%	62%	75%	None reported	61.5%	51.9%
Bifurcated (IIa)	11%	34%	5%	5%	57.7%	13.5%
Bifurcated (IIb)	20.5	34%	None reported	20%	57.7%	23.1%
Trifurcated (III)	12.3%	4%	None reported	None reported	None reported	11.5%
Presence of communication between the Pudendal nerve and the Sciatic nerve	None reported	None reported	None reported	None reported	None reported	26.9%

Mahakkanukrauh et al. (2005)reported the pudendal nerve trunking concerning the sacrospinous ligament in 37 cadavers (73 sides of hemipelves) of 21 males and 16 females, ranging from 18-83 years of age [8]. The authors elaborated to subdivide the pudendal nerve trunking into five types: type I is defined as one-trunked (41/73; 56.2%), type II is two-trunked (8/73; 11%), type III is two-trunked with one trunk as an inferior rectal nerve piercing through the sacrospinous ligament (8/73; 11%), type IV is two-trunked with one as an inferior rectal nerve not piercing through the sacrospinous ligament (7/73; 9.5%), and type V is three-trunked (9/73;12.3%) [8]. Kocabiyik et al. (2008) reported that the puden­dal nerve was defined as a single trunk in 62%, double trunk in 34%, and triple trunk in 4% [11].Pi­rro et al. (2009) reported that the pudendal nerve was a single trunk in 3/4 of the cases [12]. Maldonado et al.(2015), in a study on unembalmed female cadavers, reported that a single pudendal nerve trunk was identified in 61.5% of hemipelvis and inferior rectal nerve was noted to enter the pudendal canal in 42.3% of hemipelvis [13,14]. Shafik et al. (1995) reported that the pudendal nerve was de­rived from S2, 3, and 4 in 14/20, S1, 2, 3, and 4 in 5/20, and from S2, 3, 4, and 5 in 1/20 [6].

Mahakkanukrauh et al. (2005) reported that in case the pudendal nerve was two-trunked, the inferior rectal nerve represented one trunk piercing the sacrospinous ligament in 11% of cases or not piercing the sacrospinous liga­ment in 9.5% [8]. In our study, the two trunked pudendal nerves with one trunk as the inferior rectal nerve pierced the sacrospinous ligament in 13.5% of cases. In contrast, in 23.1% of cases, the inferior rectal nerve did not pierce the sacrospinous ligament.

Viktor Matejcik(2012) studied the pudendal nerve in 20 cadavers and found that the inferior rectal nerve penetrating the sacrospinous ligament was observed in a single case, while the inferior rectal nerve arising from the pudendal nerve before entering the pudendal canal was found in four cases [13].

O'Bichere et al. (2000), while describing a new approach for maximal exposure of pudendal nerve in 14 human cadavers for anomalies and its implications for reconstruction; reported that Type 1 (2-trunked) and Type 2 (3-trunked) of the pudendal nerve were recognized in 30% of cadavers [15].

These variations and aberrant connections can be explained within the context of embryogenic development. The variation in the pudendal nerve (root value S2, S3, and S4) and its aberrant communication with the sciatic nerve(root value L4, L5, S1, S2, and S3) are related to the embryonic development of the lower limb. At the end of the fourth week of embryonic development, limb buds start developing as an out pocket from the ventrolateral body wall by activating a group of mesenchymal cells in the somatic lateral mesoderm. The lower limb buds are visible by day 25-26 and consist of a mesenchymal core of mesoderm covered by a layer of ectoderm known as an apical ectodermal ridge (AER). Lower limb buds lie opposite the lower four lumbar and upper two sacral segments. As soon as the buds form, ventral primary rami from the appropriate spinal nerves penetrate the mesenchyme. Immediately they establish an intimate contact with the differentiating mesodermal condensations, and this early close contact between the nerve and muscle cells is essential for complete functional differentiation. These motor axons from the spinal cord enter the limb buds during the fifth week and grow into the dorsal and ventral muscle masses. Sensory axons enter the limb buds after the motor axons and use them for navigation. The spinal nerves are distributed in segmental bands, supplying both dorsal and ventral surfaces of the limbs. However, cutaneous nerve areas and dermatomes show considerable overlapping. Since many factors influence the formation of the lower limb muscles and their nerves during embryogenesis, any change may lead to variations in the innervation. Imperative variations in innervation patterns may result from altered molecular signaling between the mesenchymal cells and neuronal growth bud at the time of the fusion of the lumbosacral plexus.

The appearance of early divisions may have originated from the lack of coordination between the formation of the limb muscles and their innervation. Also, failure of differentiation may be attributed as a cause for some of the fibers taking an aberrant course into a communicating branch [16,17]. No literature or evidence can ascertain whether these are afferents or efferents. Literature was searched extensively, but no research article or case report was available on the communication between the sciatic and pudendal nerves. This finding of anomalous communication may help explain the aberrant nerve block of the sciatic or pudendal nerve when either is intentionally blocked, resulting in the blockade of both the nerves [18]. This may also be significantly useful in the diagnosis of unexplained chronic perineal or lower pelvic pain and their possible remedy by excision of such anomalous connections. Sedy et al.(2007) reported that chronic pain related to pudendal nerve entrapment is challenging to diagnose and treat accurately. Better awareness of pudendal entrapment across specialties will emerge with a better awareness of pudendal entrapment ongoing work on the subject [19]. This study may better understand pudendal neuralgia, one of the causes of chronic perineal pain. Kaur and Singh (2021) also reported that it remains primarily underdiagnosed and inappropriately treated [20].

Due to the limited sample size and lack of neurophysiological association, the results of the current study and its discussion call for cautious interpretation. Nevertheless, the observations detailed in the present study provide a comprehensive account of the extrapelvic course, variations, and connections of the pudendal nerve, which may be helpful from the clinical perspective.

## Conclusions

Henceforth, as detailed in this study, a comprehensive understanding of the course, early branching pattern, variations, and aberrant communication of the pudendal nerve is helpful for surgeons performing perineal surgeries, perirectal/perianal surgeries. Also, Improved characterization of the pudendal nerve may serve as a guide for nerve reconstruction surgery; effective pudendal nerve block will also help avoid intraoperative complications and better understand chronic perineal pain like pudendal neuralgia hence enhancing the existing treatment modalities.
